# Impact of early cleft lip and palate surgery on maxillary growth in 5- and 10-Year-old patients with unilateral cleft lip and palate: a cross-sectional study

**DOI:** 10.1186/s12903-024-05067-y

**Published:** 2024-10-29

**Authors:** Magda Novakova, Alena Brysova, Jitka Vokurkova, Petr Marcian, Libor Borak, Olga Koskova

**Affiliations:** 1 Clinic of Dentistry, St. Anne’s University Hospital Brno, Pekarska 53, Brno, 656 91 Czech Republic; 2 Department of Burns and Plastic Surgery, University Hospital Brno, Jihlavska 20, Brno, 62500 Czech Republic; 3 Faculty of Medicine, Department of Anatomy, Masaryk University, Kamenice 126/3, Brno, 625 00 Czech Republic; 4 Faculty of Medicine, Masaryk University, Brno, Czech Republic; 5 Cleft Center of the University Hospital Brno, Brno, Czech Republic; 6 Faculty of Mechanical Engineering, Institute of Solid Mechanics, Mechatronics and Biomechanics, Brno University of Technology, Technicka 2896/2, Brno, 616 69 Czech Republic; 7 Department of Pediatric Surgery, Orthopedics and Traumatology, University Hospital Brno, Cernopolni 9, Brno, 613 00 Czech Republic

**Keywords:** Neonatal cleft lip surgery, Cleft lip and palate, GOSLON, 5YO index, Dental arch relationship

## Abstract

**Objectives:**

This study evaluated maxillary growth and dental arch relationships at 5 and 10 years of age in patients with unilateral cleft lip and palate (UCLP) who underwent early cleft lip and palate surgery.

**Methods:**

28 patients with UCLP who underwent cleft lip surgery in neonatal age and cleft palate surgery at average age of 7 months without orthodontic treatment (intervention group) were measured for intercanine and intermolar distances and for dental arch length. These measurements were compared with those of 30 healthy participants in a control group. Dental arch relationships in the intervention group were evaluated by 5-YO index at 5 years and the GOSLON Yardstick score at 10 years of patients’ age.

**Results:**

Patients in the intervention group had significantly shorter mean intercanine distance and arch length than control patients at both 5 and 10 years of age (p&lt;.001 for all). There were no significant differences in intermolar distance at both 5 (*p* = .945) and 10 years (*p* = .105) of patients’ age. The average 5YO index increased from 2.46 to an average GOSLON 10-year score of 2.89 in intervention group.

**Conclusion:**

Intercanine distance and dental arch length of patients with UCLP are significantly reduced at 5 and 10 years after early cleft lip and palate surgeries compared to the healthy population. Dental arch relationships at 5 and 10 years of patients with UCLP show comparable outcomes to those reported by other cleft centers.

**Clinical significance:**

This study evaluates maxillary growth in UCLP patients 5 and 10 years of age who underwent early primary lip and palate surgery.

## Introduction

The growth of the maxilla in patients with cleft lip and palate (CLP) is influenced not only by the cleft itself but also by the surgical procedures necessary for restoring functionality and achieving aesthetic improvement. Lip reconstruction is essential to reestablish the continuity of the lip and the symmetry of the alae nasi [1]. Palate reconstruction involving the restoration of velopharyngeal closure enables improvements in swallowing, reduced occurrences of food and fluid regurgitation into the nose, decreased airway and middle ear infections, and proper development of speech [2, 3].

However, primary surgeries for the lip and palate negatively impact the growth of the maxilla, resulting in underdevelopment in the sagittal, transverse, and vertical directions [4–6]. Patients with clefts have a sufficiently wide maxilla at birth, even wider than the healthy population. It is caused by the spreading of the lateral segments by the pressure of the tongue in the intrauterine period [7]. In the postnatal period, however, growth restriction occurs. The timing of primary surgeries for patients with CLP has long been a subject of debate. Approximately 40 years ago, Bardach confirmed in rats that lip reconstruction increased pressure on the premaxilla more than in non-cleft patients [8]. While early cleft lip surgery is not generally recommended [9], there is a lack of sufficient evidence for these recommendations in humans. The previously recommended the “Rule of 10s” for timing the primary cleft lip surgery based on age, weight, and blood parameters is gradually being abandoned [10]. Nonetheless, in the Czech Republic, a trend towards neonatal cleft lip surgery has been observed since 2005 [11].

Long-term surveillance of maxillary growth in patients who underwent primary surgery at the earliest feasible age may offer novel insights into the optimal timing of primary surgeries for cleft palate. Our hypothesis is that the timing of primary cleft lip surgery does not impact maxillary growth; instead, maxillary growth is influenced by the cleft defect itself. The primary aim of this study is to compare the size of dental arches in a group of patients with unilateral cleft lip and palate (UCLP) at 5 and 10 years of age, who underwent neonatal cleft lip surgery and early cleft palate repair and have not yet undergone orthodontic treatment, with a group of healthy individuals. The secondary aim is to assess dental arch relationships in children with UCLP at 5 and 10 years of age, who underwent neonatal cleft lip surgery and early cleft palate repair, using the 5-year index [12] and at 10 years of age using the GOSLON Yardstick [13].

## Materials and methods

28 non-syndromic patients (21 boys, 7 girls) with UCLP were included in the study as the intervention group. We analyzed data obtained from the 5 and 10-year follow-ups at Orthodontic Department of Dental Clinic of St. Anne´s Hospital. Written informed consent of the participants was not needed due to the observational design of the study based on authorization by the Ethics Committee of the University Hospital St. Anne´s in Brno (Approval number for the study: EK-FNUSA-19/2022).

All patients underwent primary cleft lip surgery using the modified Millard [1, 14] technique and one-stage reconstruction of the hard and soft palate with intravelar veloplasty by one experienced plastic surgeon in Cleft Center at University Hospital Brno between years 2009 and 2012. The average age at lip surgery was 7.4 days (min-max 2–28 days after birth). Primary palatoplasty was performed at an average of 7 months and 13 days (min-max 5–12 months of age). None of the patients underwent nasoalveolar molding or any orthodontic treatment, none of them underwent alveolar bone grafting. For the control group, 30 healthy children aged 5 years and 30 healthy children aged 10 years, all without cleft and orthodontic anomalies, were selected from patients of the Pediatric department of the Dental Clinic of St. Anne’s Hospital, Brno, Czech Republic. Participants of the control group underwent a standard preventive dental examination.

Alginate impressions of the upper and lower jaw and a wax registration of the dental arch relationship were made in the intervention group at the 5-years and 10-years dispensary examination according to the recommendations of the Eurocleft study [15]. The impressions were made also in the control group.

Dental casts were made from the impressions in the laboratory and then scanned using an iTero Element 5D Plus intraoral scanner (Align Technology Inc., San Jose, CA, USA). Individual distances specified below were digitally measured on the generated 3D scans and the relationships of the dental arches were determined using OrthoCAD software (Align Technology Inc., San Jose, CA, USA). Distances were measured to the nearest hundredth of a millimeter. All measurements were performed twice with an interval of at least one week. For the dental arch characteristics, average values from the two measurements were used for the assessment.

**Fig. 1:**
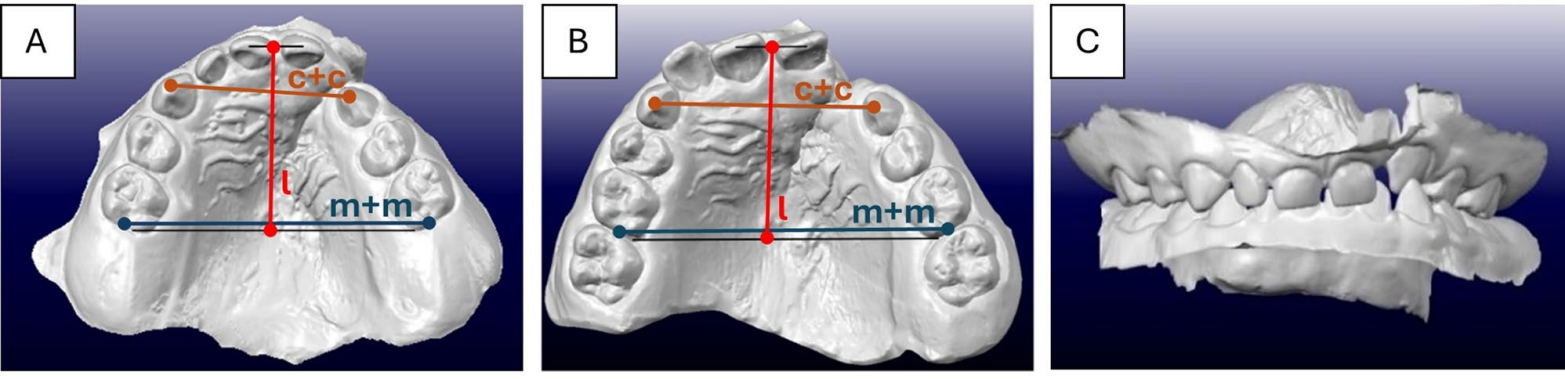
Analysis of intercanine distance, intermolar distance and arch length. **A**, 3D scan of unilateral cleft lip and palate at 5 years old patient. **B**, 3D scan of unilateral cleft lip and palate at 10 years old patient. **C**, dental arch relationship of the jaws. c + c = intercanine distance, m + m = intermolar distance, l = arch length

Specifically, the following distances were measured on each 3D scan of maxilla (Fig. 1):

1. The intercanine distance (c + c) was measured from the cuspid of the upper canine on the left side to the cuspid of the upper canine on the right side [16].
2. The intermolar distance (m + m) was measured from the center of the distal surface of the second deciduous molar on the left side to the center of the distal surface of the second deciduous molar on the right side [17, 18].
3. The length (l) of the upper dental arch was measured from the point between the upper incisors, perpendicular to the junction of the distal approximal surfaces of the second deciduous molars [16].

The most commonly used method for evaluation of the dental arch relationships in children with UCLP is the GOSLON Yardstick Index [13]. This method is used for both mixed and permanent dentition of 10-years old patients. Based on this method, Atack suggested a 5-year index (5YO index) for assessments in the deciduous dentition of 5-years old patients [12]. The GOSLON Yardstick and the 5YO indices categorize the occlusal outcome into one of five categories from excellent (Category 1) to very poor (Category 5). Specifically, the sagittal, vertical and transverse relationships of the jaws are assessed with Category 1 indicating a normal relationship of the dental arches. On the contrary, Category 5 means deficient condition and indicates underdevelopment of the entire zygomaticomaxillary complex. The intermediate relationships are categorized as good, fair or poor. The dental arch relationships were assessed independently by two raters, each performing the assessment twice (i.e. in two sessions) with an interval of at least one week.

### Statistical analysis

Quantitative data were expressed using mean, standard deviation (SD), median, minimum and maximum values. Shapiro-Wilk normality tests showed that all measurements of dental arch characteristics followed a normal distribution. Two-sample paired t-tests were used for the comparison of dependent samples (from intervention group only), and two-sample unpaired t-tests for independent samples (from both intervention and control groups). The non-parametric Wilcoxon Signed-Ranks Test was used to compare results of dental arch relationship (based on 5YO and GOSLON indices). The following three null hypotheses were tested: There are no significant statistical differences (1) between intervention and control groups in terms of dental arch characteristics, (2) between 5- and 10-year-old patients in the intervention group in terms of dental arch characteristics and (3) between 5- and 10-year-old patients in the intervention group in terms of 5YO and GOSLON indices. All tests were performed at the 0.05 significance level.

Intra-rater reliability of GOSLON and 5YO indices evaluations was assessed using Cohen’s kappa. This statistic measures the extent to which one rater repeatedly evaluate the same phenomenon consistently. Inter-rater reliability was examined using the Fleiss Multi-Rater Kappa which is an extension of Cohen’s kappa for more than two rating sessions (here two raters each evaluating twice). McHugh [19] suggested interpreting the Kappa results as follows: values ≤ 0.20 indicate no agreement, 0.21–0.39 minimal agreement, 0.40–0.59 weak agreement, 0.60– 0.79 moderate agreement, 0.80–0.90 strong agreement, and above 0.90 almost perfect agreement. In general, any kappa below 0.60 can be interpreted as an inadequate agreement.

The statistical software IBM SPSS Statistics for Windows, Version 23.0 was used for statistical processing. Armonk, NY: IBM Corp. and the MedCalc v18.2 program (MedCalc Software, Ostend, Belgium).

## Results

At 5 years, patients with UCLP had a statistically significantly lower mean intercanine distance (intervention: 26.7 mm, control: 30.2 mm, *p* < .001) and shorter arch length (intervention: 26.8 mm, control: 30.1 mm, *p* < .001) than control patients. On the contrary, there was no statistically significant alteration observed in the intermolar distance (intervention: 42.1 mm, control: 42.2 mm, *p* = .945). Detailed comparisons are provided in Table 1.

**Table 1:** Dental arch characteristics in cleft patients and control patients at 5 years (in milimeters), p-values of two-sample unpaired t-test

Distance	Intervention group, 5-years old (*n* = 28)					Control group, 5-years old (*n* = 30)					*p*-value
	Mean	SD	Median	Min	Max	Mean	SD	Median	Min	Max	
c + c	26.67	3.01	26.35	22.25	32.65	30.16	2.37	29.80	25.55	34.60	< 0.001
m + m	42.14	3.14	42.35	32.,05	46.95	42.20	2.89	41.43	37.20	47.90	0.945
l	26.78	2.29	26.83	22.80	30.45	30.14	1.42	30.23	27.60	33.10	< 0.001

At 10 years, there were again statistically significant differences in the intercanine distance (intervention: 27.5 mm, control: 33.5 mm, *p* < .001) as well as in the length of the dental arch (intervention: 25.9 mm, control: 31.6 mm, *p* < .001). No statistically significant difference in the intermolar distance between cleft patients and the control group was observed (intervention: 42.4 mm, control: 43.5 mm, *p* = .108). Details are provided in Table 2.

**Table 2:** Dental arch characteristics in cleft patients and control patients at 10 years (in milimeters), p-values of two-sample unpaired t-test

Distance	Intervention group, 10-years (*n* = 28)					Control group, 10-years (*n* = 30)					*p*-value
	Mean	SD	Median	Min	Max	Mean	SD	Median	Min	Max	
c + c	27.48	3.83	26.90	21.90	35.15	33.46	1.51	33.18	31.10	36.70	< 0.001
m + m	42.39	3.15	42.25	36.60	48.70	43.54	2.18	43.98	38.00	48.20	0.108
l	25.88	3.47	26.45	15.40	31.70	31.57	2.06	31.35	27.60	35.70	< 0.001

When comparing the maxillary morphology of cleft patients between 5 and 10 years of age, pairwise tests showed that only the intercanine distance increased statistically significantly (intervention: 26.7 mm, control: 27.5 mm, *p* = .007). There is a significant change also in the arch length (intervention: 26.8 mm, control: 25.9 mm, *p* = .032); however, in this case, a decrease of the measured distance was observed with the age. The distance between molars did not change significantly (intervention: 42.1 mm, control: 42.4 mm, *p* = .527). See Table 3 for the details.

**Table 3:** Dental arch characteristics in cleft patients in 5 years and in 10 years (in milimeters), p-values of two-sample paired t-test

Distance	Intervention group, 5-years (*n* = 28)					Intervention l group, 10-years (*n* = 28)					*p*-value
	Mean	SD	Median	Min	Max	Mean	SD	Median	Min	Max	
c + c	26.67	3.01	26.35	22.25	32.65	27.48	3.83	26.90	21.90	35.15	0.007
m + m	42.14	3.14	42.35	32.05	46.95	42.39	3.15	42.25	36.60	48.70	0.527
l	26.78	2.29	26.83	22.80	30.45	25.88	3.47	26.45	15.40	31.70	0.032

**Fig. 2:**
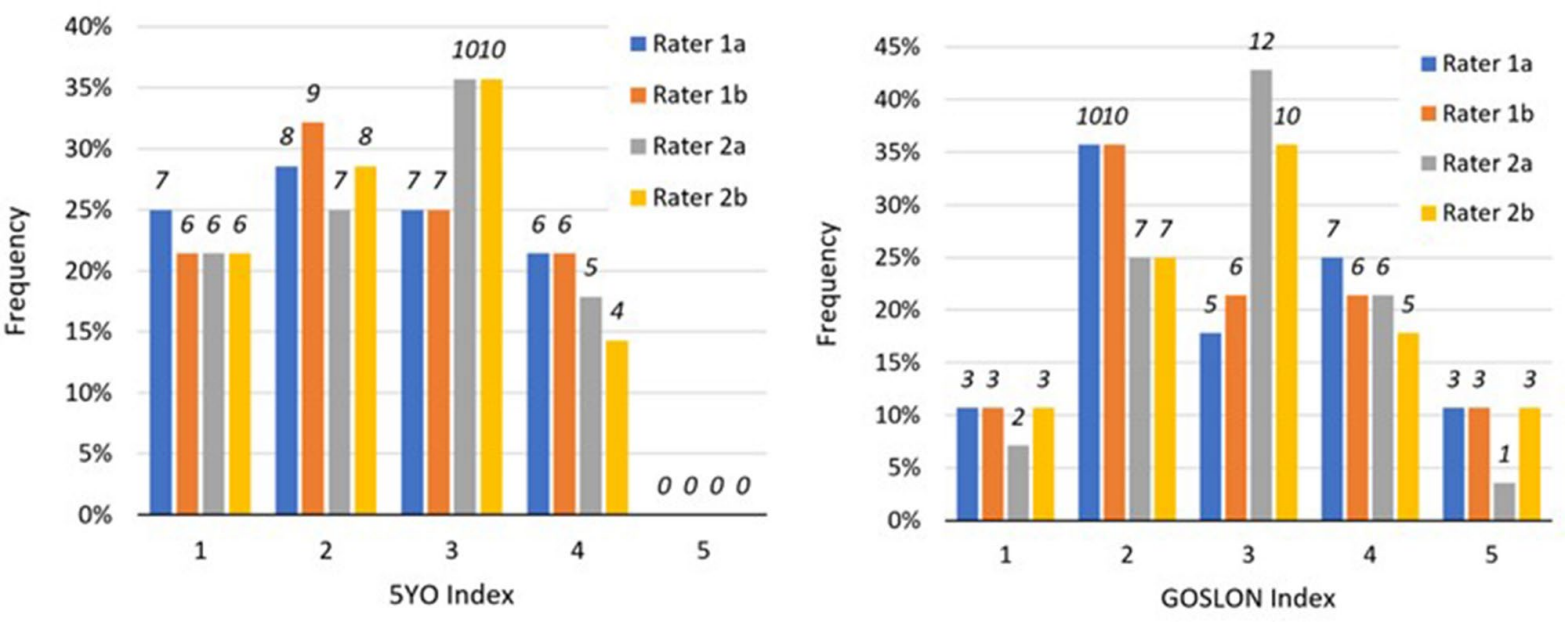
Analysis of dental arch relationship: Relative frequency in % (absolute frequency is shown above each bar). **A**, Frequency of 5YO indices in cleft patients. **B**, Frequency of GOSLON indices in cleft patients

In UCLP patients without orthodontic treatment, between 5 and 10 years of age, there was a statistically significant deterioration in the evaluation of the dental arch relationship according to the Wilcoxon Signed Rank Test applied to 5YO and GOSLON indices (*p* = .001 through 0.017). The average 5YO index in 28 patients without orthodontic treatment increased from 2.46 to an average GOSLON 10-year score of 2.89 (Table 4). Frequencies of 5YO and GOSLON indices as evaluated by both raters during both evaluation sessions are shown in Fig. 2.

**Table 4:** 5YO and GOSLON indices in cleft patients measured by 2 raters, each in 2 sessions, p-value of Wilcoxon test

Index	Mean	p-value			
		Rater 1a	Rater 1b	Rater 2a	Rater 2b
5YO	2.46	0.001	0.005	0.017	0.002
GOSLON	2.89				

Intra-rater agreement on 5YO and GOSLON indices evaluations using Cohen’s kappa for both raters was rated as strong to almost perfect (Rater #1: κ = 0.97 and 95% CI 0.91−1.00 for 5YO, κ = 0.97 and 95% CI 0.92−1.00 for GOSLON; Rater #2: κ = 0.94 and 95% CI 0.85−1.00 for 5YO, κ = 0.84 and 95% CI 0.72−0.96 for GOSLON). Inter-rater agreement using the Fleiss Multi-Rater Kappa was rated in both cases as moderate (5YO: κ = 0.77 and 95% CI 0.68−0.86; GOSLON: κ = 0.73 and 95% CI 0.65−0.82).

## Discussion

Cleft defects are a diverse group of birth defects that manifest themselves in many different clinical symptoms. Comparative studies, therefore, contain very small patient samples. Each center has its own treatment protocols that differ, both in terms of operative technique and timing. It also depends on the experience of the multidisciplinary cleft team [15].

Current studies indicate a tendency towards ever earlier timing of surgery, whether it is lip reconstruction in newborns [9, 20, 21], palatoplasty in first year [2, 3] (previously performed only at 2–3 years) or secondary bone grafting, which is planned according to teeth eruption [22]. The timing of cleft lip surgery depends on the practices of the cleft center, special pediatric equipment and an experienced pediatric anesthesiologist are necessary for cleft lip surgery in the neonatal period. Historically, the “Rule of 10s” was recommended as a guideline for timing cleft lip surgery (at least 10 weeks of age or older, a weight of 10 pounds, a hemoglobin exceeding 10 g/dL, and a white blood cell count < 10,000/mm³). However, due to advancements in pediatric anesthesia and surgical techniques, this concept is gradually being abandoned [10, 23]. Recent data show that early lip surgery, from an anesthesia perspective, does not carry greater risks compared to standard timing [24]. Early, or neonatal, cleft lip surgery offers significant benefits for improving mother-child interaction after birth [25]. The widely debated negative impact of early anesthesia on psychosocial development and IQ in children with clefts has not yet been proven [26]. On the contrary, several studies have been published supporting the idea of improved feeding following early lip surgery [27, 28]. Some authors also highlight the importance of persistent fetal healing in the early postnatal period and the better maturation of lip scars [29]. The timing of palatoplasty is also still a subject of discussion. The assessment of the effects of different timings of primary surgeries on jaw growth has already been described, except for the impact of neonatal cleft lip reconstruction, which has not yet been published in detail. Ross’s study [30] of cephalometric images of 15 cleft centers did not confirm any significant differences in the effect on further growth. Studies of adult patients with unoperated complete unilateral cleft lip and palate [31] describe a normal potential for maxillary growth.

Our results show a significant difference in the intercanine distance and dental arch length in patients with UCLP between the ages of 5 and 10, confirming a smaller intercanine distance and shorter arch length compared to normal patients. However, the palatal width in the intermolar distance was found to be similar to that of the control group. These findings align with the general understanding that, in patients with UCLP after reconstruction of the lip and palate, the growth of the upper jaw is typically underdeveloped in the sagittal, transverse, and vertical directions [4–6]. As a result, we frequently encounter skeletal class III malocclusions and pseudoprogeny, where the maxilla is underdeveloped while the mandible is of normal size. In some cases, the mandible may exhibit slightly larger growth due to the lack of growth restriction from overbite. This often leads to anterior crossbite, lateral segment crossbite, and a concave facial profile [32, 33]. The reduced transverse and sagittal dimensions of the dental arch in these patients before orthodontic treatment and alveolar bone grafting, as supported by other studies [17, 34], underscore the importance of early intervention strategies aimed at addressing these maxillary deficiencies. Our findings further highlight the need for precise treatment planning to mitigate the long-term skeletal and dental impacts associated with UCLP.

Many options can be used to evaluate the overall treatment of UCLP, such as the relationship of dental arches [12, 13], the size of dental arches [17, 34], cephalometric radiographs [17, 35] or CBCT [36, 37]. A small number of studies describe the condition of dental arches in patients with clefts who have not undergone orthodontic treatment. In most cases, the 5YO index is used to evaluate the surgery performed and the GOSLON score to evaluate the results between cleft centers. The 5YO index in untreated patients ranged from 2.41 to 2.96 in previously published data [35, 38, 39]. In our study, the mean value of the 5YO index was 2.46, which we can consider as a good agreement.

In 2019, Peterson [40] published a large retrospective study of the GOSLON Index, comparing the average GOSLON Index with 28 other international studies. It should be noted, however, that if we compare at 10-years-old patients who have been orthodontically treated and untreated, the result will be very variable. From our point of view, it seems important to define orthodontic treatment and to compare the GOSLON index at 10 years in similarly treated patients. For this reason, only patients not treated orthodontically were included in the study and compared with other two previously published studies [41, 42]. In the first study, Susami [42] selected 24 patients with total unilateral cleft lip and palate aged 7–10 years who had not undergone any orthodontic treatment or alveolar bone grafting and underwent primary cleft lip surgery within the first 6 months of age, and the palate closure within the first 2 years of age. Susami noted that almost 60% of the children were categorized as 4 or 5, and the average GOSLON index was 3.5. In the second study, Southall [41] divided a total of 66 patients who underwent primary cleft lip surgery at 3 months and primary palate closure between 6 and 9 months of age into two groups. In the first group, there were 47 orthodontically untreated patients included with total unilateral cleft, whose mean index was 3.17. The second group consisted of patients with a cleft defect who were orthodontically treated and the GOSLON index was 2.16. In the present study, only 25-36% (depending on the rater) of the 28 patients were enrolled in categories 4 and 5 and the mean GOSLON score was 2.89, which we consider as a very good agreement.

## Limitations

The number of patient samples was limited in this study. Nowadays, there is an effort to initiate orthodontic therapy and adjust the dental arches earlier, resulting in fewer patients who have not undergone any orthodontic therapy [38, 43].

It is also necessary to consider the fact that the patients without treatment who were included in the present study were those not very interested in active orthodontic treatment, either for social reasons or because of the child’s or parent´s compliance. We also observed a higher percentage of carious and untreated teeth in these patients, making it impossible to start treatment with an orthodontic appliance.

In cases of carious dentition, due to premature tooth extraction, the perimeter of the dental arch is reduced; therefore, the intermolar distance and the length of the palate can be reduced as well because of the mesial inclination and/or displacement of the molars [44].

## Conclusion

The intercanine distance and the length of the dental arches are statistically significantly reduced at 5 and 10 years in patients with UCLP after neonatal lip reconstruction and primary palatoplasty up to one year of age compared to the healthy population. The intermolar distance neither at 5 nor at 10 years was changed compared to the healthy population. Between the ages of 5 and 10 years, there was a significant increase in the intercanine distance, but it still does not reach the values of the healthy population.

More favorable results were achieved in the 5-Year-Old Index and Goslon Index in our study compared to previously published studies that also included only non-orthodontically treated patients, but where the primary surgeries were performed at a later timing. This may indicate that the timing of primary cleft lip and palate surgeries may not be a decisive factor in maxillary development. However, these findings require further validation in a larger cohort of patients, including an evaluation of dentoalevolar relationships after complete growth. However, it would be beneficial to verify these conclusions after the final completion of jaw growth in the presented group of patients.

## Data Availability

All data generated or analyzed during this study are included in this published article, further inquiries are available from the corresponding author on reasonable request.

## Abbreviations

UCLP: Unilateral Cleft Lip and Palate
5YO: 5-YO index
CLP: Cleft Lip and Palate
SD: Standard Deviation
